# *Macrobrachium rosenbergii* nodavirus infection in a giant freshwater prawn hatchery in Indonesia

**DOI:** 10.1186/s40064-016-3127-z

**Published:** 2016-10-06

**Authors:** Murwantoko Murwantoko, Arif Bimantara, Roosmanto Roosmanto, Masashi Kawaichi

**Affiliations:** 1 Fish Diseases Laboratory, Department of Fisheries, Faculty of Agriculture, Universitas Gadjah Mada, Jl Flora Bulaksumur, Yogyakarta, 55281 Indonesia; 2 Yogyakarta Giant Freshwater Prawn Hatchery, Ikoma, Japan; 3 Division of Gene Function in Animals, Nara Institute of Science and Technology, Ikoma, Japan

**Keywords:** *Macrobrachium rosenbergii*, MrNV, Nodavirus, RNA viruses, White tail disease

## Abstract

A pathogen of giant freshwater prawn, *Macrobrachium rosenbergii*, was recently recorded in a hatchery in Yogyakarta. The clinical symptom in post-larvae (PL) was a whitish appearance of the muscles in the tail. Histological examination revealed myonecrosis with massive infiltration of myonuclei and hemocytes. RT-PCR products of 850 bp were obtained when using RNA from diseased PL as a template. The clinical signs and RT-PCR amplicon were reproduced in *M. rosenbergii* inoculated with bacteria-free inocula. Electron microscopy demonstrated that the *M. rosenbergii* nodavirus (MrNV) was icosahedral in shape and 28.12 ± 2.31 nm in diameter. RT-PCR products of the RNA-dependent RNA polymerase gene (RNA-1) and capsid protein gene (RNA-2) of MrNV were obtained using designed primer pairs, cloned into pBluescript-KS, and sequenced. The 1312 nucleotide (nt) sequence of MrNV RNA-1 revealed 98.0 % identity with isolates from China and India. Additionally, the 1112 nt sequence of MrNV RNA-2 displayed 98.0 % identity with isolates from China and Taiwan. Disease control efforts involving disinfection of PL, broodstocks, water media, tanks, equipment and ponds successfully eradicated white tail disease from the hatchery. This study is the first report on white tail disease and the isolation and characterization of MrNV in Indonesia.

## Background

The giant freshwater prawn, *Macrobrachium rosenbergii* (de Man), is a common inhabitant of rivers and estuaries in tropical regions of the world (New 2005). The giant freshwater prawn is distributed in almost all islands in Indonesia. Since 1990, this prawn has been considered an important commodity and is cultured in West Java, Central Java, Yogyakarta, East Java and Bali (Nugroho and Emmawati 2004; Nugroho et al. 2005). In order to meet the demand for freshwater prawn fry, hatcheries have been developed in Yogyakarta,West Java and in Bali (Nugroho and Emmawati 2004). Aquaculture statistic from Ministry Marine Affairs and Fisheries showed that in 2013 the prawn has been produced also at Banten, Aceh, West Borneo, South Borneo.

Declining growth rate, disease and the decreasing size of the edible portion of the prawn are the main problems for freshwater prawn in Indonesia (Nugroho and Emmawati 2004). One way to increase production is through the genetic improvement program. In 2001, the GI Macro (Genetically Improved *Macrobrachium rosenbergii*), strain of freshwater prawn has been developed and released by Ministry Marine Affair and Fisheries. The GI Macro prawns have been distributed to three hatcheries in East Java; Yogyakarta; and West Java (Nugroho et al. 2005).

A viral disease on giant freshwater prawn, white tail disease (WTD) or white muscle disease (WMD), was first reported in the French West Indies (Arcier et al. 1999), then in China (Qian et al. 2003), India (Sahul Hameed et al. 2004), Thailand (Yoganandhan et al. 2006), Taiwan (Wang et al. 2008) and Australia (Owens et al. 2009). This disease caused 100 % mortality within 2 or 3 days in freshwater prawn hatcheries and nursery ponds in different parts of India (Sahul Hameed et al. 2004). A 50 % production loss in more than 50 freshwater prawn hatcheries situated in the affected states alone has caused an economic loss of about US$15 million annually. Further losses in grow-out farm production because of poor survival of the PL with a low-level asymptomatic infection could result in severe economic losses.

The main clinical sign of WTD is a whitish coloration of muscles, starting in some areas of the tail, extending to the tail muscles (abdomen) and, at the final stage, extending to all the muscles of the prawn. These signs are associated with abnormal behavior, lethargy and anorexia. When investigated histologically, lesions are found in muscle and connective tissues. These lesions correspond to small dense basophilic inclusions, 0.5–3.0 µm in diameter, located in the cytoplasm. The muscles were necrotic and muscle fibers were dissociated (Arcier et al. 1999). The causative agent of WTD has been identified as *Macrobrachium rosenbergii* nodavirus (MrNV) associated with extra small virus (XSV) (Qian et al. 2003). MrNV is a small icosahedral non-enveloped virus and is 26–27 nm in diameter. The viral genome is composed of two fragments of linear, single-stranded, positive-sense RNAs (RNA-1 and RNA-2) of approximately 2.9 and 1.3 kb, respectively. The viral capsid contains a single polypeptide of 43 kDa (Bonami et al. 2005).

Vertical transmission of MrNV and XSV in M. rosenbergii has been confirmed and been thought as main mechanism of the disease transmission (Sudhakaran et al. 2007). In horizontal transmission experiments, five developmental stages of artemia were exposed to MrNV and XSV by immersion and oral routes concluded that artemia acts as a reservoir or carrier of the viruses (Sudhakaran et al. 2006a). Artificial infection on *Macrobrachium malcolmsonii* and *M. rude* with *Mr*NV and XSV by oral and intramuscular routes did not produce mortality or clinical symptoms, however those prawns could act as reservoir for *Mr*NV and XSV and maintaining their virulence in their tissues (Ravi and Sahul Hameed 2014). Another animals also can act as a reservoir for MrNV and XSV such as redclaw crayfish (Cherax quadricarinatus) Hayakijkosol et al. (2011), marine shrimps *Penaeus indicus*, *P. japonicus*, and *P. monodon* (Sudhakaran et al. 2006b), *P. vannamei* (Senapin et al. 2012). Not only shrimp, the aquatic insects, the giant water bug Belostoma sp., dragonfly nymphs Aesohna sp., diving beetles Cybister sp. and back swimmers Notonecta sp may present a risk for MrNV and XSV transmission (Sudhakaran et al. 2008).

In December 2011, GI Macro freshwater prawn larvae from a number of tanks in a giant freshwater hatchery in Samas Yogyakarta displayed whitish tails followed by death. This study examined the causal agent of this disease and the control effort in the hatchery. This study is the first report on white tail disease and the isolation and characterization of MrNV in Indonesia.

## Results and discussion

### Clinical signs and histology

**Fig. 1:**
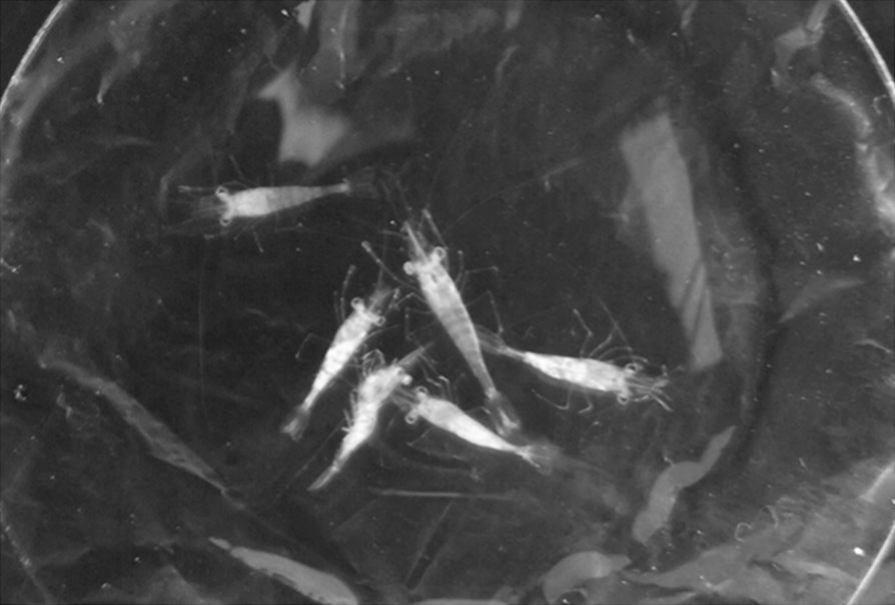
Post-larvae of *Macrobrachium rosenbergii* with different levels of whitish tails

**Fig. 2:**
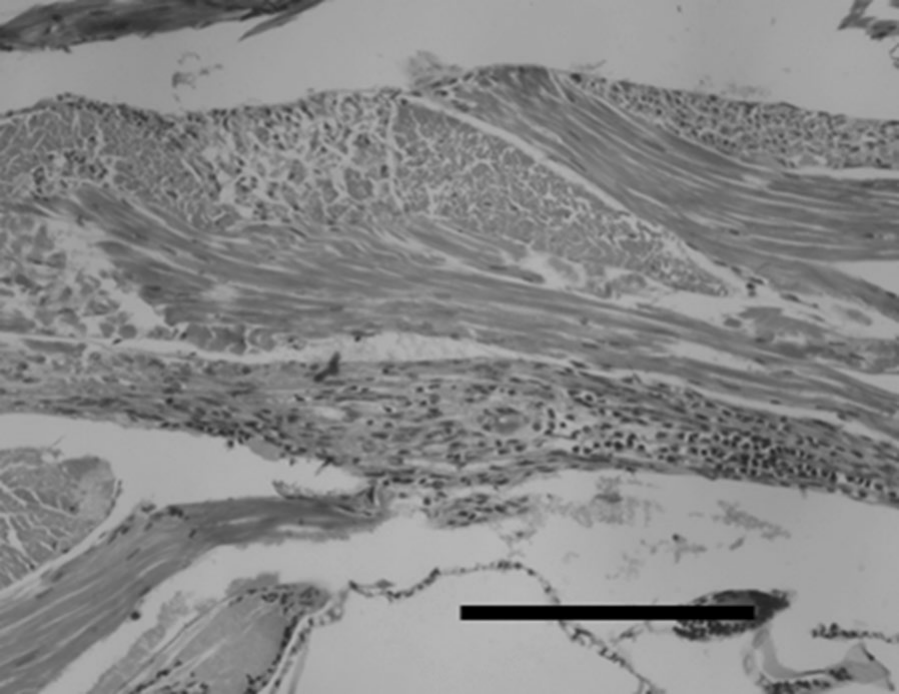
Infiltration of hematocytes in the tail of Macrobrachium rosenbergii post-larvae (*scale bar* 250 µm)

In December 2011, the giant freshwater prawn hatchery in Samas Yogyakarta reported the mortality of post-larvae (PL). The gross appearance of those PL showed prominent whitish muscles in the abdominal region. Different levels of PL color could be observed, ranging from the normal transparent, faint white band to nearly complete white coloration across the entire abdomen (Fig. 1). The number of white tail PL increased over the subsequent days of rearing and was followed by increased mortality. Mass mortality occurred 4–6 days after the first white tail PL appeared. Histological analysis of those PL displayed several stages of infiltration of myonuclei and some hemocytes on tail muscles. Limited infiltration could be seen on dorsal muscles and massive infiltration in ventral muscles (Fig. 2). Extensive myonecrosis and dissociation of muscle fibers were also observed (data not shown).

### Infectivity experiment

**Fig. 3:**
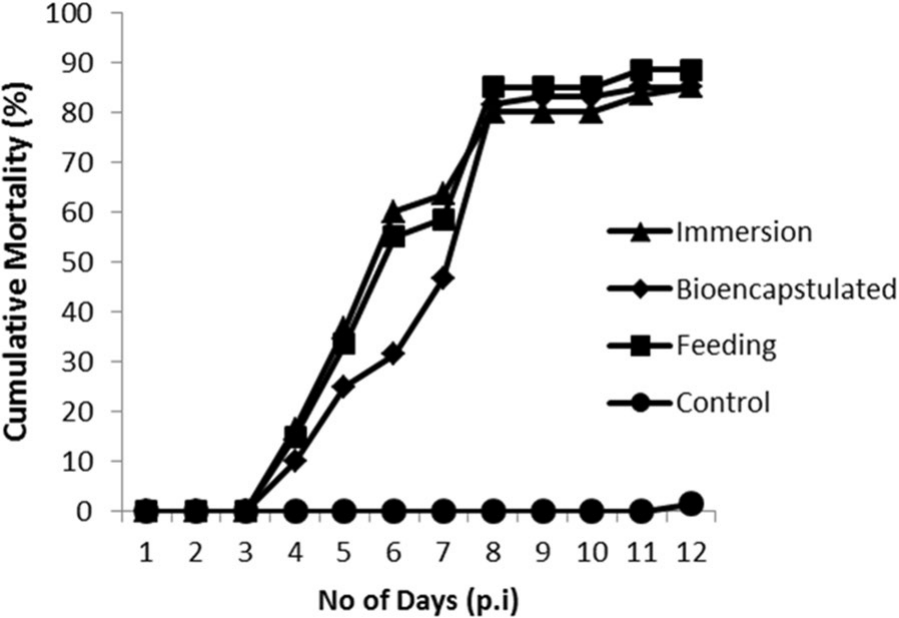
Cumulative percent mortality of post-larvae of *Macrobrachium rosenbergii* infected by feeding with infected PL, immersion and feeding with inoculum bioencapsulated-artemia. The control PL were reared without inoculum immersion and fed with untreated artemia

To verify the disease agents and their transmission, an infectivity test was performed by separate treatments of feeding with infected PL, immersion, and feeding with inoculum bioencapsulated-artemia. The white color of the abdomen segment appeared 3 days after all infection treatments and was followed by death at 4 days post-infection. When the experiment was stopped at 12 days, the control prawn PL had the lowest mortality (1.5 %), whereas the infection via immersion and bioencapsulated-artemia treatments had high mortality (85 %), and the fed with diseased PL treatment had the highest mortality (88.5 %). The cumulative mortality of the infection treatments was significantly higher (P < 0.01) compared to the control, and no significant difference in cumulative mortality was observed between infection treatments (Fig. 3).

### RT-PCR

**Fig. 4:**
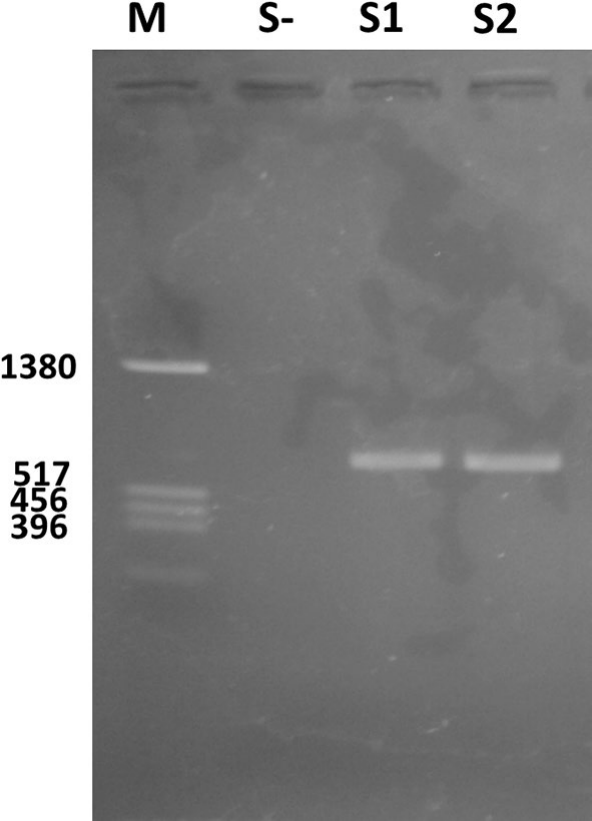
Agarose gel showing reverse transcription-polymerase chain reaction products of *Macrobrachium rosenbergii* nodavirus (MrNV) amplified using the described primer pairs. An 850 bp amplified product was obtained from sample 1 (*S1*) and sample 2 (*S2*) from diseased PL but was not obtained from the non-template control (*S*-). M: marker DNA with indicated size

To verify the presence of virus in giant freshwater prawn PL, RNA was extracted from PL and subjected to reverse transcription PCR. The presence of a single band of 850 bp was detected by RT-PCR (Fig. 4). The death and moribund PLs from the infectivity experiments were collected and their RNA was purified. RT-PCR analysis demonstrated that RNA from moribund PL also produced an 850 bp band, whereas healthy PL did not produce a band (data not shown).

### Purification and electron micrograph of virus

**Fig. 5:**
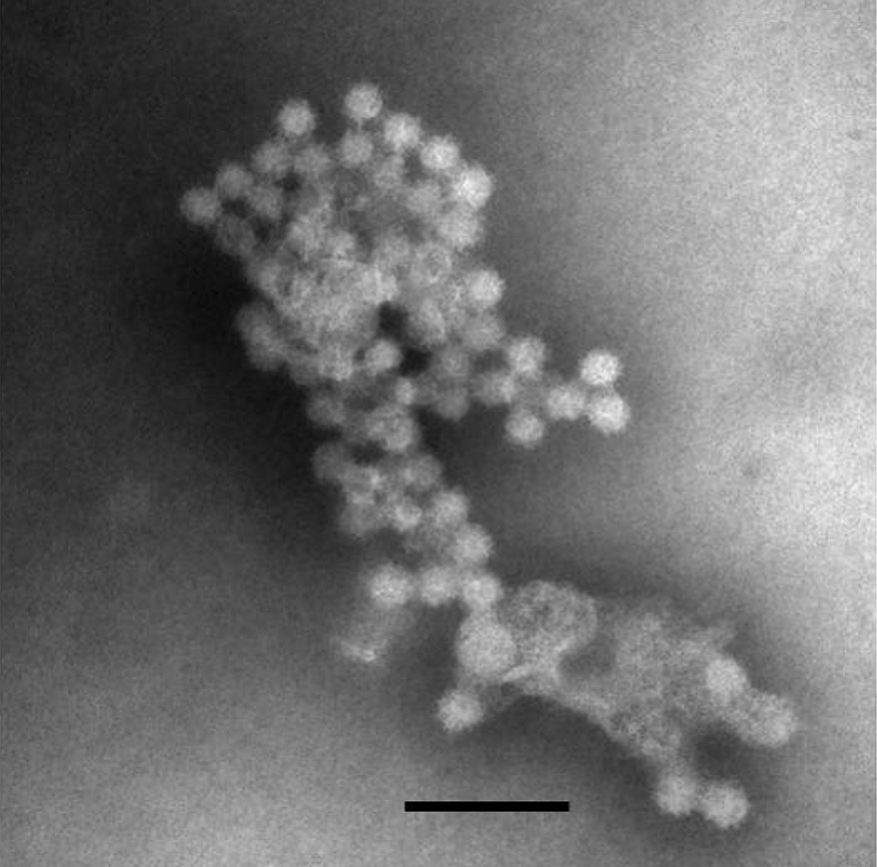
Transmission electron micrographs of *Macrobrachium rosenbergii* nodavirus (MrNV). (2 % PTA, *scale bar* 100 nm)

Disease PL were homogenized and clarified, and the virus suspension was then subjected to sucrose gradient ultracentrifugation. After centrifugation, no clear band was visible. However, analysis of absorbance at 260 and 280 nm revealed that some fractions contained a significant number of particles. Those fractions were pelleted and examined by electron microscopy. After negative staining, TEM analysis indicated that small particles were present in the fractions. In the higher density fraction, five- and six-sided particles were observed, indicating an icosahedral structure. The diameter of the particles was 28.12 ± 2.31 nm (n = 30) (Fig. 5).

### Sequence analysis

In this study, a primer pair was designed to amplify capsid protein gene and another pair was designed to amplify the RdRP gene of MrNV. Those primer pairs were successfully used to amplify the genetic material of MrNV, as demonstrated by a single band that appeared after RT-PCR. RT-PCR using MrNV-CP-F/MrNV-CP-R and MrNV-RdRP-2F/MrNV- RdRP-2R primers produced DNA fragments of 1100 and 1300 bp in size, respectively. Ligation reactions between a plasmid vector and these amplicons were transformed into Escherichia coli DH 5α. Numerous colonies appeared on LB agar containing ampicilin. Colony PCR for several colonies demonstrated that almost all colonies contained the insert fragment DNA. Those results indicated that the amplified DNAs were successfully cloned into pBluescript-KS.

Sequencing of the recombinant plasmids demonstrated that the cloned DNA fragments are 1312 nt corresponding to 3′-terminal end of MrNV RdRP gene (RNA-1) and 1112 nt corresponding to the coding region of MrNV capsid protein gene (RNA-2). BLAST analysis of the cloned RNA-1 revealed 98.0 % identity to the MrNV RdRP gene from China (accession FJ751226) and from India (accession JQ18295). Analysis of cloned RNA-2 revealed 98.0 % identity to the viral capsid protein gene (RNA-2) of MrNV from Taiwan (accession DQ 521575) and from China (accession FJ751225). The Indonesian MrNV RNA-1 and RNA-2 sequences are stored in Genbank with accession numbers KF824533 and KF824532, respectively.

**Fig. 6:**
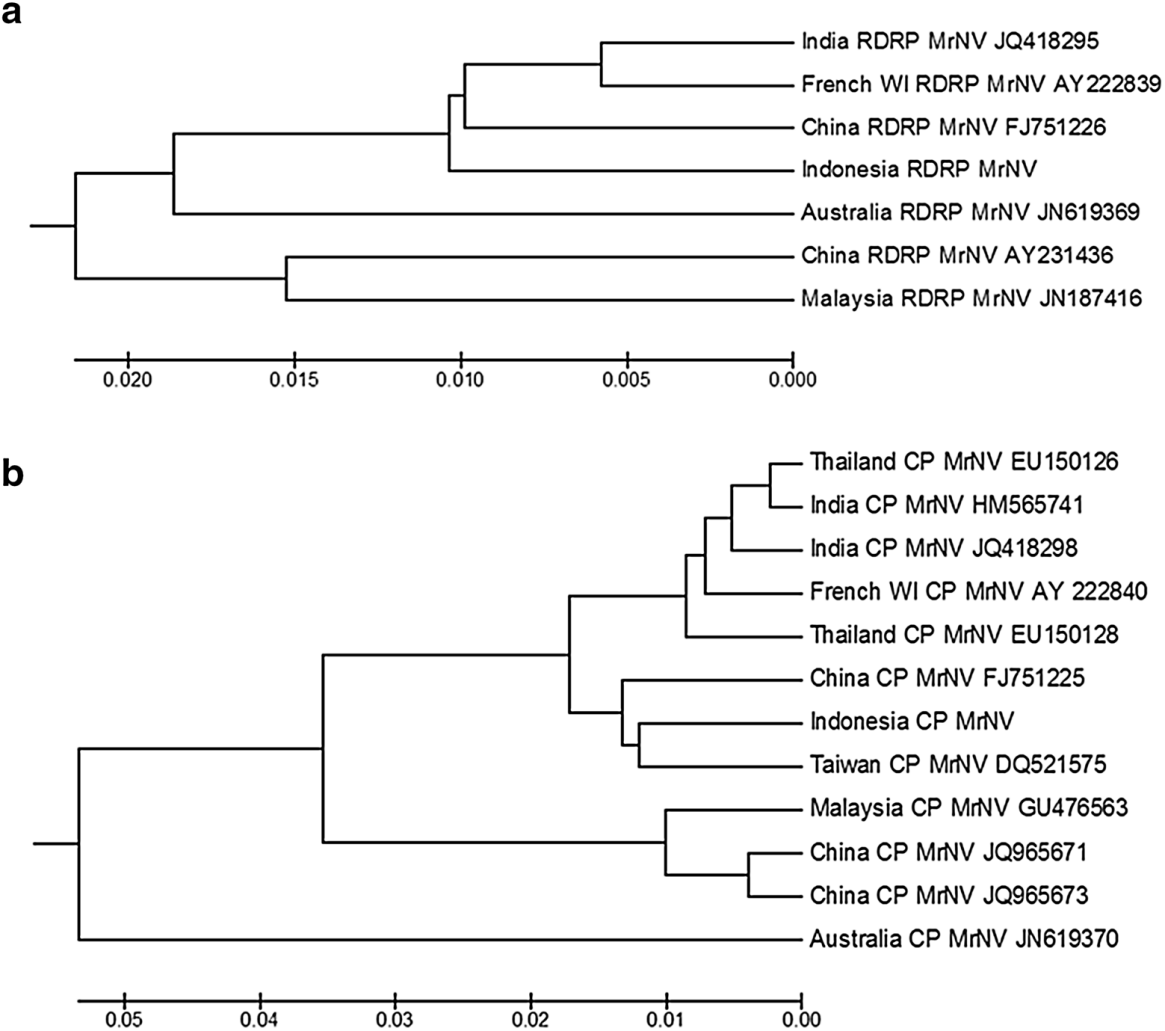
Phylograms of the sequence of the Indonesia isolate of *Macrobrachium rosenbergii* nodavirus (MrNV) compared with other isolates of MrNV using both the 1312 nt sequences from the RdRP gene (**a**) and the 1112 nt sequences of the capsid protein gene (**b**)

The phylograms of MrNV using capsid protein and RdRP genes indicated the diversity of the virus, and the RdRP gene appeared more diverse compared with the capsid protein gene. From those phylograms, based on Unweighted Pair Group Method with Arithmetic Mean (UPGMA) analysis of the sequences, the MrNV Indonesian isolate is most closely related to the MrNV isolate from China (accession FJ751226, FJ751225) (Fig. 6).

### Disease control

When this disease appeared and mortality occurred, the PL produced by the hatchery on December 2011 were not distributed to the farmer but were instead kept in hatchery tanks. When the disease was verified as being caused by the virus, the PLs, water, tanks and equipment were disinfected using 300 ppm calcium hypochloride. Tanks and equipment were further sterilized by fumigation with potassium permanganate. RT-PCR analysis also performed to screen for the presence of MrNV in the other aquatic animals in the hatchery area. This analysis demonstrated that broodstocks of giant freshwater prawn were also infected by MrNV. The virus could be detected in hemolymph, muscles and eggs of the broodstocks (data not shown). The broodstocks were disinfected by burning, liming and burying on March 22, 2012. The broodstock rearing ponds were disinfected by liming and drying.

## Discussion

Since 1990, giant freshwater prawn has been considered an important commodity and is cultured In order to meet the demand for freshwater prawn fry, hatcheries have been developed in Yogyakarta,West Java and in Bali (Nugroho and Emmawati 2004). Yogyakarta giant freshwater prawn hatchery is most important giant freshwater prawn hatcheries in Indonesia. The production capacity of Yogyakarta hatchery was the highest with approximately 11 million PL/year, then Bali hatchery with approximately 7 million PL/year and West Java hatcehry with approximately 300,000 PL/year. The economic value from the Yogyakarta hatchery was approximatey USD 7.7 million annualy (Nugroho et al. 2005). The Yogyakarta hatchery not only produced PL to be cultured in Yogyakarta Province but also to be cultured in West Java, Central Java and East Java Provinces, Borneo island.

A white tail disease of giant freshwater prawn post-larvae, *Macrobrachium rosenbergii*, was recorded on December 2011 at Yogyakarta giant freshwater prawn hatchery. The gross clinical signs and histopathology indicated that this disease has similarity with white tail disease (WTD) (Arcier et al. 1999; Owens et al. 2009). Infection experiments indicated that the disease could be transmitted in several ways. This disease can be transmitted by cannibalism, and giant freshwater prawns exhibit a high degree of cannibalism. Bacteria-free inoculum can caused disease in PL both by media immersion or through artemia as a vector. An RT-PCR assay of naturally and laboratory-infected PL verified that *M. rosenbergii nodavirus* (MrNV) was present in those PL. The clinical signs and RT-PCR assay results fulfilled River’s postulates and confirmed the presence of white tail disease caused by MrNV. This study is the first report regarding white tail disease in Indonesia. WTD has been observed in the French West Indies (Arcier et al. 1999), China (Qian et al. 2003), India (Sahul Hameed et al. 2004), Thailand (Yoganandhan et al. 2006), Taiwan (Wang et al. 2008) and Australia (Owens et al. 2009).

Ultrastructural analysis of the higher density fraction revealed that the icosahedral virus with a diameter of 28.12 ± 2.31 nm is MrNV. These data are consistent with the previous study showing that MrNV is an icosahedral virus with a diameter of 27 nm (Bonami et al. 2005). The genetic variability of MrNV could be observed from BLAST analysis and the phylogram, however the variability is not large. BLAST analysis of RNA-2 of MrNV from Asian countries showed that among 19 sequences that have 100 % query coverage, 14 exhibited at least 96 % identity and another 5 have 94 % identity. The Australian isolate was a little different from the Asian isolates, displaying 92 % identity. Based on UPGMA analysis of the RNA-1 and RNA-2 sequences, the Indonesian isolate of MrNV is most closely related to the MrNV isolate from China (accession FJ751226, FJ751225) (Fig. 6), which was originally collected from Hainan Province of China in 2006 (Senapin et al. 2012).

To control this disease in the giant freshwater prawn hatchery, the PL, water, broodstocks were disinfected, and all tanks and equipment also were sterilized. The broodstock rearing ponds were disinfected by liming and drying. We believed that those treatments could eradicate potential vector or reservoir for disease transmission from tanks and ponds such as artemia, insects, crustaceans. After hatchery sterilization, MrNV-free larvae were introduced to produce PL. Pond-reared broodtsocks were obtained from selected MrNV-free juvenile giant freshwater prawnsMrNV-free juvenile giant freshwater prawns were introduced to be reared to be broodstocks. Between March 2012 and July 2015, the hatchery produced 20 batches of PL. No MrNV infection was detected by RT-PCR analysis in those batches of PL (data not shown). These results indicated that control efforts have successfully eradicated WTD from the hatchery.

## Conclusions

This study confirmed the white tail disease on giant freshwater prawn, *Macrobrachium rosenbergii*, from a hatchery in Yogyakarta. M. rosenbergii nodavirus (MrNV) was characterized as icosahedral in shape and 28.12 ± 2.31 nm in diameter. The RNA-dependent RNA polymerase gene (RNA-1) and capsid protein gene (RNA-2) of this MrNV showed 98.0 % identity with isolates from China and India, and 98.0 % identity with isolates from China and Taiwan respectively. Disease control efforts involving disinfection of PL, broodstocks, water media, tanks, equipment and ponds successfully eradicated white tail disease from the hatchery.

## Methods

### Animal collection

Uninfected and infected post-larvae (PL) of *M. rosenbergii* with prominent signs of whitish muscle in the abdominal and tail regions were collected from a hatchery in Samas Yogyakarta. These animals were transported to the laboratory in oxygenated plastic bags.

### Histology

Twenty PL were placed in Davidson’s fixative for 48 h. After fixation, tissues were transferred to 70 % ethanol, processed for histology using standard methods and embedded in paraffin wax. Five micrometer sections were cut and stained with Mayer’s hematoxylin and eosin. Light microscopy (Olympus EC microscope) was used to observe the sections. Photographs were taken using a Panasonic digital camera.

### Infectivity experiment

The PL of *M. rosenbergii* with whitish muscles in their abdominal regions were selected and used as inoculum for infectivity experiments with healthy post-larvae. Frozen PL were thawed and homogenized in a sterile homogenizer. A 10 % (w/v) suspension was made with TN buffer (20 mM Tris–HCl and 0.4 M NaCl, pH 7.4). The homogenate was centrifuged at 4000×*g* for 5 min at 4 °C and its supernatant was centrifuged at 10,000×*g* for 5 min at 4 °C. Then, the final supernatant was filtered through a 0.22-μm pore membrane. The filtrate was stored at −20 °C for infectivity studies.

Twenty uninfected PL were placed in each of 5 L beakers containing freshwater with continuous aeration. The beakers were covered to prevent contamination. The post-larvae were fed artemia nauplii. The infectivity experiment was performed in triplicate with the following treatments: feeding with infected PL, adding inoculum into the rearing media (immersion) and feeding with bioencapsulated inoculum artemia. In the feeding with infected PL treatment, the healthy PL were fed with infected PL for one day, followed by artemia. In the immersion treatment, the inoculum prepared as mentioned above was added to the water at a volume equal to 0.1 % of the total rearing medium (1 ml/L). Artemia bioencapsulation was performed by adding the inoculum into a beaker containing artemia in freshwater with continuous aeration for 1 h. The artemia were washed extensively with freshwater. In the bioencapsulation treatment, the inoculum-bioencapsulated artemia were fed to healthy PL for one day. Control groups were PL without infection and fed with artemia.

The numbers of deaths were recorded and the cumulative mortality levels were calculated. Cumulative mortality was assayed using analysis of variance; the mean mortality between treatments was determined by least significant difference (Gomes and Gomes 1984). The PL at moribund stages were collected, and RT-PCR was performed to confirm the presence of MrNV.

### Reverse transcriptase-polymerase chain reaction (RT-PCR)

PL or tissues (100 mg) were homogenized in 300 μl of TN buffer and centrifuged at 10,000×*g* for 5 min. The supernatants were used to extract RNA using the High Pure RNA isolation kit (Roche, Mannheim, Germany) following the manufacturer’s protocol. For detection of MrNV from PL, RT-PCR was performed using the Transcriptor One-Step RT-PCR Kit (Roche, Mannheim, Germany) using the forward primer 1A775 (CCACGTTCTTAGTGGATCCT) and the reverse primer 1B690 (CGTCCGCCTGGTAGTTCC). The primers were designed to amplify RNA-dependent RNA polymerase of MrNV at 850 bp in size (Sri Widada et al. 2003). Reactions were performed in 25 μl RT-PCR solution containing 20 pmol of each primer and RNA template using the following steps: RT at 52 °C for 30 min; denaturation at 95 °C for 2 min followed by 30 cycles of denaturation at 94 °C for 30 s, annealing at 55 °C for 30 s and elongation at 72 °C for 1 min, ending with an additional elongation step of 10 min at 72 °C. The RT-PCR products (10 μl) were then analyzed by electrophoresis on a 1 % agarose gel stained with ethidium bromide and visualized using an ultraviolet transilluminator.

### Purification of viral particles

Approximately 500 mg of PL were homogenized in TN buffer and clarified at 1.400×*g* for 15 min using a JA 25.50 Beckman rotor in a Beckman Avanti J25 centrifuge. Supernatant was collected and a second clarification was performed at 10,000×*g* for 15 min. For pelleting, the supernatant was then centrifuged at 150,000×*g* for 4 h in a Ti 70 rotor in a Beckman ultracentrifuge L8-70M. After re-suspension in TN buffer, the final pellet was layered onto 15–30 % (w/w) sucrose in a TN buffer gradient and run at 135,000×*g* for 3 h using an SW 40 Ti rotor. The 0.5 ml fractions were collected from the gradient manually. The presence of viruses in fractions was examined using a spectrophotometer (Beckman DU-600) at 260 nm and 280 nm. The desired fractions were diluted in TN buffer, pelleted at 150,000×*g* for 4 h in a Ti 70 rotor and finally suspended in water.

### Transmission electron microscopy

We examined the morphology of the purified viruses by transmission electron microscopy using colloid carbon coated grids (300 mesh) (Nisshin EM). Virions were fixed in 4 % PFA and negatively stained with 2 % PTA. Virions were observed in a Hitachi H 7100 electron microscope operating at 75 kV. Virion size was analyzed by measuring the diameter of 30 particles.

### Cloning

For cloning, primers were designed based on data from Genbank for capsid protein (CP) gene (accession number GU300102), RNA-dependent RNA polymerase (RdRP) gene (accession number JN187416.1) using Oligocalculator software (http://www.basic.northwestern.Edu/biotools/oligocalc.html). The following primers to amplify CP and - RdRP genes: MrNV-CP-F (CTCCGAGAATTCATGGCTAGAGGTAAACAAA) and MrNV-CP-R (TGTCCCTGGATCCACAACCTAATTATTGCCGAC) for RdRP, and MrNV- RdRP-2F (AGGGAATTCTTGTGACTATGTTCGTGG) and MrNV-RdRP-2R (AGGTTCGAAGCTTAGCAATGGTAACTC) for CP. The EcoRI, BamHI or HindIII restriction enzyme site was added at the 5′ terminal end of the primer. Amplification of the DNA was performed by RT-PCR as explained above.

The amplicons were purified using phenol–chloroform followed by digestion with the appropriate enzyme. After digestion, the PCR products were electrophoresed in agarose and the DNA bands were purified. Purified amplicons were ligated into pBluescript-KS (Stratagene) that was digested by the same restriction enzymes using T4 DNA ligase (Toyobo) at 16 °C for overnight. Ligation mixtures were transformed into Escherichia coli DH5α using heat shock at 42 °C for 90 s followed by incubation on ice. The bacteria were cultured on LB agar plates containing 50 µg/ml ampicilin overnight. The presence of recombinant plasmids in colonies was checked by colony PCR using the designed primers. The colonies containing inserts were cultured in LB broth containing ampicilin and at 37 °C overnight. The plasmids were isolated from the bacteria using minipreparation of the alkali lysis method (Sambrook and Russel 2001).

### Sequencing and DNA analysis

The recombinant plasmids were purified using the PEG/NaCl method and sequenced using T3 and T7 primers with Big Dye terminator v3.1 (Applied Biosystems). The PCR products were applied to an ABI310 sequencer. DNA sequences from those two primers were overlapped to determine the complete sequence of the inserted DNA fragments. DNA sequences were analyzed using BLAST (Altschul et al. 1990). The phylogenetic tree based on several DNA sequences in GenBank was constructed using the UPGMA method in Mega 5.2 software.

## References

[CR1] Altschul SF, Gish W, Miller W (1990). Basic local alignment search tool. J Mol Biol.

[CR2] Arcier JM, Herman F, Lightner DV (1999). A viral disease associated with mortalities in hatchery-reared post-larvae of the giant freshwater prawn *Macrobrachium rosenbergii*. Dis Aquat Org.

[CR3] Bonami JR, Shi Z, Qian D (2005). White tail disease of the giant freshwater prawn, *Macrobrachium rosenbergii*: separation of the associated virions and characterization of MrNV as a new type of nodavirus. J Fish Dis.

[CR4] Gomes KA, Gomes AA (1984). Statistical procedures for agricultural research.

[CR5] Hayakijkosol O, La Fauce K, Owens L (2011). Experimental infection of redclaw crayfish (*Cherax quadricarinatus*) with *Macrobrachium rosenbergii* nodavirus, the aetiological agent of white tail disease. Aquaculture.

[CR6] New MB (2005). Freshwater prawn farming: global status, recent research and a glance at the future. Aquacult Res.

[CR7] Nugroho E, Emmawati L (2004). Giant freshwater prawn culture in Indonesia.

[CR8] Nugroho E, Sugama K and Maskur (2005) Genetic improvement of *Macrobrachium rosenbergii* in Indonesia. Report of the second round table discussion on the development of genetically improved strain of Macrobrachium. SEAFDEC, pp 4–8

[CR9] Owens L, Fauce KL, Juntunen K (2009). *Macrobrachium rosenbergii* nodavirus disease (white tail disease) in Australia. Dis Aquat Org.

[CR10] Qian D, Shi Z, Zhang S (2003). Extra small virus-like particles (XSV) and nodavirus associated with whitish muscle disease in the giant freshwater prawn, *Macrobrachium rosenbergii*. J Fish Dis.

[CR11] Ravi M, Sahul Hameed AS (2014). Experimental transmission of *Macrobrachium rosenbergii* nodavirus (*Mr*NV) and extra small virus (XSV) in *Macrobrachium malcolmsonii* and *Macrobrachium rude*. Aquacult Int.

[CR12] Sahul Hameed AS, Yoganandhan K, Sri Widada J (2004). Studies on the occurrence of *Macrobrachium rosenbergii* nodavirus and extra small virus-like particles associated with white tail disease of *M. rosenbergii* in India by RT-PCR detection. Aquaculture.

[CR13] Sambrook J, Russel DV (2001). Molecular cloning: a laboratory manual.

[CR14] Senapin S, Jaengsanong C, Phiwsaiya K (2012). Infections of MrNV (*Macrobrachium rosenbergii* nodavirus) in cultivated white leg shrimp *Penaeus vannamei* in Asia. Aquaculture.

[CR15] Sri Widada J, Durand S, Cambournac I (2003). Genome-based detection methods of *Macrobrachium rosenbergii* nodavirus, a pathogen of the giantfreshwater prawn, *Macrobrachium rosenbergii*: dot-blot, in situ hybridisation and RT-PCR. J Fish Dis.

[CR16] Sudhakaran R, Yoganandhan K, Ishaq Ahmed VP (2006). Artemia as a possible vector for *Macrobrachium rosenbergii* nodavirus (MrNV) and extra small virus transmission (XSV) to Macrobrachium rosenbergii postlarvae. Dis Aquat Org.

[CR17] Sudhakaran R, Syed Musthaq S, Haribabu P (2006). Experimental transmission of *Macrobrachium rosenbergii* nodavirus (MrNV) and extra small virus (XSV) in three species of marine shrimp (*Penaeus indicus*, *Penaeus japonicas* and *Penaeus monodon*). Aquaculture.

[CR18] Sudhakaran R, Ishaq Ahmed VP, Haribabu P (2007). Experimental vertical transmission of *Macrobrachium rosenbergii* nodavirus (MrNV) and extra small virus (XSV) from brooders to progeny in *Macrobrachium rosenbergii* and Artemia. J Fish Dis.

[CR19] Sudhakaran R, Haribabu P, Rajesh Kumar S (2008). Natural aquatic insect carriers of Macrobrachium rosenbergii nodavirus (MrNV) and extra small virus (XSV). Dis Aquat Org.

[CR20] Wang CS, Chang JS, Wen CM (2008). *Macrobrachium rosenbergii* nodavirus infection in M. rosenbergii (de Man) with white tail disease cultured in Taiwan. J Fish Dis.

[CR21] Yoganandhan K, Leartvibhas M, Sriwongpuk S (2006). White tail disease of the giant freshwater prawn Macrobrachium rosenbergii in Thailand. Dis Aquat Org.

